# Novel Combinations of Agents Targeting Translation That Synergistically Inhibit Fungal Pathogens

**DOI:** 10.3389/fmicb.2018.02355

**Published:** 2018-10-04

**Authors:** Cindy Vallières, Roxane Raulo, Matthew Dickinson, Simon V. Avery

**Affiliations:** ^1^School of Life Sciences, University of Nottingham, University Park Campus, Nottingham, United Kingdom; ^2^School of Biosciences, University of Nottingham, Sutton Bonington Campus, Loughborough, United Kingdom

**Keywords:** antifungal combinations, amino acids, synergistic fungicides, plant pathogens, fungal disease, crop protection

## Abstract

A range of fungicides or antifungals are currently deployed to control fungi in agriculture or medicine, but resistance to current agents is growing so new approaches and molecular targets are urgently needed. Recently, different aminoglycoside antibiotics combined with particular transport inhibitors were found to produce strong, synergistic growth-inhibition of fungi, by synergistically increasing the error rate of mRNA translation. Here, focusing on translation fidelity as a novel target for combinatorial antifungal treatment, we tested the hypothesis that alternative combinations of agents known to affect the availability of functional amino acids would synergistically inhibit growth of major fungal pathogens. We screened 172 novel combinations against three phytopathogens (*Rhizoctonia solani*, *Zymoseptoria tritici*, and *Botrytis cinerea*) and three human pathogens (*Cryptococcus neoformans*, *Candida albicans*, and *Aspergillus fumigatus*), showing that 48 combinations inhibited strongly the growth of the pathogens; the growth inhibition effect was significantly greater with the agents combined than by a simple product of their individual effects at the same doses. Of these, 23 combinations were effective against more than one pathogen, including combinations comprising food-and-drug approved compounds, e.g., quinine with bicarbonate, and quinine with hygromycin. These combinations [fractional inhibitory combination (FIC) index ≤0.5] gave up to 100% reduction of fungal growth yield at concentrations of agents which, individually, had negligible effect. No synergy was evident against bacterial, plant or mammalian cells, indicating specificity for fungi. Mode-of-action analyses for quinine + hygromycin indicated that synergistic mistranslation was the antifungal mechanism. That mechanism was not universal as bicarbonate exacerbated quinine action by increasing drug uptake. The study unveils chemical combinations and a target process with potential for control of diverse fungal pathogens, and suggests repurposing possibilities for several current therapeutics.

## Introduction

A wide range of fungi are undesirable for different reasons, including human and plant pathogens. Pathogenic fungi of humans, such as the ascomycetes *Candida albicans* and *Aspergillus fumigatus*, and the basidiomycete *Cryptococcus neoformans*, cause life-threatening infections particularly for individuals with compromised immunity. Invasive fungal infections have mortality rates between 20 and 95% and are estimated to cause ∼1.5 million deaths annually, comparable to the mortality associated with tuberculosis and malaria (Brown et al., 2012; Calderone et al., 2014; Denning and Bromley, 2015). Compared to antibacterial treatments, the current arsenal of approved agents for the treatment of fungal infections is limited, with only four classes of antifungal compound targeting different structures or pathways. Their efficacy is eroded by toxicity or drug resistance (Vandeputte et al., 2012; Delarze and Sanglard, 2015; Van Dijck et al., 2018). The paucity of current treatments can be explained partly by the common eukaryotic nature of fungal pathogens and their hosts. Therefore, finding fungal-specific agents is challenging. In addition, a number of fungi are major plant pathogens, with the potential to devastate food crops (Cools and Hammond-Kosack, 2013; Oliver and Hewitt, 2014). Reflecting this, the global fungicide market is worth more than $7 billion annually. A wide range of fungicides have been developed and approved to counter these phytopathogens. However, the agrichemical industry also faces concerns surrounding resistance emergence combined with tightening of fungicide regulations (Oliver and Hewitt, 2014; Coste and Vandeputte, 2015), underscoring the urgent need for new effective treatments for fungal control.

Combination treatments to control fungal growth provide an attractive strategy for management. Such combinations often allow decreased effective-doses versus single agent usage, especially where the agents act synergistically, and this can lower the costs and potential for toxicity (Johnson et al., 2004; Zimmermann et al., 2007; Spitzer et al., 2017). For example, the current gold standard antifungal treatment for cryptococcal meningitis is a combination of 5-flucytosine and amphotericin B. A mixture of mancozeb and copper hydroxide, two agents with multi-site contact activity, is used in agriculture for protection from fungal diseases. Moreover, combinatorial inhibition can slow the evolution of resistance. Another advantage of such combinations is that they may allow existing, approved drugs to be repurposed (in novel combination treatments), bypassing the costly and time-consuming development of new drugs or fungicidal agents.

Strong synergistic interactions between potential antifungals are not very common. Detection of novel synergies can be facilitated by screening approaches and/or the use of a good model system. The yeast *Saccharomyces cerevisiae* is widely deployed as a eukaryotic cell model, with an advanced molecular toolbox. Such advantages have been applied to characterize mechanisms of antifungal drug action and find synergistic antifungal combinations (Islahudin et al., 2013; Moreno-Martinez et al., 2015; Robbins et al., 2015). In one study, a strong, synergistic inhibition of yeast growth was noted when the aminoglycoside antibiotic paromomycin was combined with chromate (Holland et al., 2007). This example of synergy has been further considered for its antifungal potential (Moreno-Martinez et al., 2015). It was shown that paromomycin plus chromate also caused strong growth-inhibition of several human pathogens and phytopathogens. Furthermore, these agents could be substituted with alternative aminoglycosides or sulfate-transport inhibitors, respectively, to achieve synergistic inhibition (Moreno-Martinez et al., 2015). Aminoglycosides cause errors in the process of mRNA translation, i.e., mistranslation (Carter et al., 2000; Fan-Minogue and Bedwell, 2008). In the case of sulfate-transport inhibitors, it was shown that: (i) cysteine and methionine starvation could mimic Cr-induced translation errors, (ii) genetic suppression of S starvation suppressed Cr-induced mistranslation, and (iii) mistranslation required cysteine and methionine biosynthesis (Holland et al., 2010). The inferred mechanism of synergy was that sulfate limitation, caused by chromate or other sulfate mimetic, decreases the supply of S containing amino acids needed for translation, so synergizing with the ribosome-targeting aminoglycosides (Moreno-Martinez et al., 2015; Vallieres and Avery, 2017a). Therefore, these studies revealed a combination treatment that appeared to act on translation fidelity, a novel potential target for antifungal action.

In the present study, we considered a much more diverse range of compound classes with the potential to affect availability of amino acids for protein synthesis. Depletion of particular amino acids within cells can alter the competition between cognate and non-cognate aminoacyl-tRNAs, leading to mistranslation (Gallant and Lindsley, 1998; Farabaugh and Bjork, 1999; Sorensen, 2001). We hypothesized that the alternative compound classes could also target translation-fidelity synergistically when supplied in appropriate combinations. Out of the 172 novel combinations screened here, 48 were found to be effective in suppressing growth of fungal phytopathogens and/or human pathogens. Discovery of novel synergistic combinations could offer a panel of alternatives to help realize stability, toxicity, cost or resistance advantages in antifungal applications, while helping toward understanding of the molecular bases for synergistic mistranslation observed here.

## Materials and Methods

### Strains, Culture, and Maintenance

Pathogenic organisms used in this study were the yeasts *C. albicans* SC5314 and *C. neoformans* 1841, the filamentous fungi *A. fumigatus* CBS 144.89, *Botrytis cinerea* SAR109940, *Rhizoctonia solani* AG2-1 1939 and *Zymoseptoria tritici*, and the bacterium *Pseudomonas aeruginosa* PA-W1 (kindly provided by Miguel Camara, University of Nottingham, United Kingdom). Mode of action studies were performed with *S. cerevisiae* BY4743 (*MAT*a/α *his3*Δ*1/his3*Δ*1 leu2*Δ*0/leu2*Δ*0 LYS2/lys2*Δ*0 met15*Δ*0/MET15 ura3*Δ*0/ura3*Δ*0*). The yeasts were maintained and grown in YPD medium [2% peptone (Oxoid, Basingstoke, United Kingdom), 1% yeast extract (Oxoid), 2% D-glucose], or YNB medium [0.69% yeast nitrogen base without amino acids (Formedium, Norfolk, United Kingdom), 2% D-glucose, supplemented with amino acids or nucleobases as appropriate for plasmid selection (Ausubel et al., 2007)]. The filamentous fungi were routinely maintained and grown on potato dextrose agar or broth [PDA (Oxoid) or PDB (Sigma-Aldrich)] except *A. fumigatus* which was on Aspergillus complete medium (ACM) (Paoletti et al., 2005) and *B. cinerea*, on Vogel’s medium (Vogel, 1964) Where necessary, media were solidified with 2% agar (Sigma-Aldrich, St. Louis, MO, United States).

### Chemicals

With the exception of hygromycin B (PanReac Applichem), and ziram and thiram (provided by Taminco BVBA), all drugs were from Sigma-Aldrich. Stock solutions of chemicals used in this study were prepared in water, DMSO, HCl or ethanol as solvent (**Table 1**). Aliquots were added to growth media as required to give the specified final concentrations.

**Table 1:** Compound concentrations used in this study.

Compounds	Solvent	*R. solani*	*B. cinerea*	*S. tritici*	*A. fumigatus*	*C. neoformans*	*C. albicans*	*P. aeruginosa*	Human TE671
Sodium chromate	Water	10 μM	10 μM	10 μM	75 μM	12.5 μM	25 μM	ND	ND
Sodium molybdate	Water	1 mM	1 mM	1 mM	30 mM	1 mM	10 mM	ND	ND
Sodium orthovanadate	Water	200 μM	2 mM	250 μM	15 mM	1 mM	250 μM	3 mM	40 μM
Sodium oxalate	Water	5 mM	5 mM	25 μM	3 mM	5 mM	5 mM	ND	ND
Sodium malonate dibasic	Water	20 mM	20 mM	10 mM	30 mM	30 mM	30 mM	ND	ND
Probenecid	DMSO	10 μM	10 μM	10 μM	1.5 mM	1 mM	1 mM	ND	ND
Sodium bicarbonate	Water	1.25 mM	20 mM	500 μM	30 mM	10 mM	25 mM	30 mM	30 mM
Sodium selenate	Water	500 μM	500 nM	100 μM	100 μM	50 μM	30 mM	30 mM	75 μM
Thiram technical	DMSO	10 ng/ml	5 ng/ml	10 ng/ml	750 ng/ml	100 ng/ml	500 ng/ml	ND	ND
Ziram technical	DMSO	50 ng/ml	1 ng/ml	25 ng/ml	750 ng/ml	100 ng/ml	500 ng/ml	3 μg/ml	25 ng/ml
Mancozeb	DMSO	75 ng/ml	50 ng/ml	100 ng/ml	5 μg/ml	100 ng/ml	750 ng/ml	ND	ND
Quinine hydrochloride dihydrate	70% EtOH	1 mM	1 mM	500 μM	2 mM	250 μM	2.5 mM	2.5 mM	75 μM
Eugenol	70% EtOH	100 μM	100 μM	100 μM	1 mM	125 μM	600 μM	3 mM	500 μM
Copper(II) sulfate pentahydrate	Water	75 μM	1 mM	75 μM	750 μM	5 mM	5 mM	1.5 mM	
Cyprodinil	70% EtOH	10 μM	1 nM	200 nM	40 μM	25 μM	50 μM	2 mM	750 nM
3,4-Dihydroxy-L-phenylalanine (DOPA)	1 M HCl	425 μM	1 mM	425 μM	1 mM	1 mM	5 mM	ND	ND
DL-Ethionine	1 M HCl	10 μM	200 nM	500 μM	500 μM	1 mM	5 mM	ND	ND
DL-Norvaline	Water	15 mM	15 mM	20 mM	20 mM	20 mM	20 mM	20 mM	20 mM
Streptomycin sulfate salt	Water	15 mg/ml	5 mg/ml	1 μg/ml	10 mg/ml	500 μg/ml	35 mg/ml	ND	ND
Paromomycin sulfate salt	Water	25 μg/ml	50 μg/ml	500 ng/ml	10 mg/ml	12.5 μg/ml	200 μg/ml	50 μg/ml	1 mg/ml
Hygromycin B	Water	250 ng/ml	500 ng/ml	250 ng/ml	15 μg/ml	625 ng/ml	10 μg/ml	40 μg/ml	50 μg/ml
Quinacrine dihydrochloride	Water	ND	100 μM	ND	ND	ND	1.5 mM	ND	ND
Mefloquine hydrochloride	70% EtOH	ND	ND	ND	ND	ND	200 μM	ND	ND
Primaquine bisphosphate	Water	ND	100 μM	ND	ND	ND	1 mM	ND	ND

*Concentrations listed (except those in red) were selected as the highest concentrations of the individual compounds which allowed growth of the indicated microorganisms, or metabolic activity of TE671 cells, at a rate that did not differ significantly from the control rate. In red, maximum concentrations that could be used while ensuring either that the added volume from stock solution was ≤3% of final volume, or to preclude inhibition by HCl solvent; these concentrations may be lower than just-subinhibitory.*

### Growth Inhibition and Toxicity Assays

Yeast growth inhibition assays were performed with broth cultures in 48-well microtiter plates, with continuous shaking at 30°C in a BioTek Powerwave XS microplate spectrophotometer, as described previously (Moreno-Martinez et al., 2015). *P. aeruginosa* was tested in a similar way but with growth in 96-well plates (Greiner Bio-one; Stonehouse, United Kingdom), in LB broth and at 37°C. Spores of *A. fumigatus* were inoculated from ACM plates to ACM broth (15,000 spores ml^-1^). Aliquots (150 μl) of the spore suspension plus any chemical supplements, as specified, were transferred to 96-well plates and cultured statically over 48 h at 37°C, with OD_600_ measured daily in a BioTek EL800 microplate spectrophotometer. *B. cinerea* (10,000 spores ml^-1^), and *Z. tritici* (20,000 spores ml^-1^) and *R. solani* (1 mg ml^-1^ of mycelium) were prepared as above in Vogel’s or PDB media, respectively, and cultured over several days at 24°C, 120 rev. min^-1^. Human cells (TE671, rhabdomyosarcoma RD cell line) were cultured in DMEM (Sigma) supplemented with 10% fetal bovine serum, 2 mM L-glutamine, 100 U ml^-1^ penicillin, 100 μg ml^-1^ streptomycin, in 25 cm^2^ cell culture flasks, 36.5°C, 5% oxygen. Toxicity to the TE671 cells was assayed using the CCK-8 reagent (Sigma) as described previously (Moreno-Martinez et al., 2015). To assess plant toxicity, the compounds were sprayed onto the surface area of lettuce leaves and the average surface of resultant lesions was measured after 3 days using ImageJ.

For assaying antifungal combinations, concentrations of agents used were those determined from preliminary assays to be just sub-inhibitory or slightly inhibitory when supplied individually. For yeast, growth (OD_600_) was monitored every 30 min over at least 18 h. Exponential-phase growth rates were calculated and expressed as percentage inhibition relative to control growth in the absence of added inhibitors.

For filamentous fungi, culture densities (OD_600_) determined after 48 h (*A. fumigatus*) or 4–7 days (plant pathogens) were also expressed in terms of percentage inhibition relative to control growth.

Screen hits were considered as those compounds showing a percentage inhibition when in combination that was greater by 20% than the sum of inhibitory effects observed when the compounds were supplied individually.

### Checkerboard Assays

All culturing and preparation for checkerboard assays adhered to EUCAST guidelines (Rodriguez-Tudela et al., 2008; Arendrup et al., 2012), with the exception that media and cell concentrations were the same as for the growth inhibition assays to ease comparison. Briefly, culture aliquots were transferred to 96-well microtiter plates with chemicals added as specified. The inoculated plates were incubated statically for 24 h (*S. cerevisiae* and *C. albicans*), 48 h (*C. neoformans* and *A. fumigatus*) or 4–7 days (plant pathogens) under either ambient air conditions or anaerobic conditions [(10% CO_2_, 10% H_2_, and 80% N_2_) in a Whitley DG250 Anaerobic Workstation (Don Whitley Scientific)]. OD_600_ was then measured with a BioTek EL800 microplate spectrophotometer. Fractional inhibitory concentration (FIC) as an indicator of synergy was calculated as described (Hsieh et al., 1993). To test whether combinations were fungicidal or fungistatic, 4 μl of cell suspension (*S. cerevisiae*) treated as above were transferred to YPD agar and incubated for 24 h at 30°C to determine colony forming ability.

### Biofilm Inhibition Assays

Biofilm metabolic activity was measured by the XTT (tetrazolium salt, 2,3-bis[2-methyloxy-4-nitro-5-sulfophenyl]-2H-tetrazolium-5-carboxanilide) (Sigma) reduction assay (Zago et al., 2015). Single yeast colonies were used to inoculate YPD broth cultures in Erlenmeyer flasks and incubated at 37°C with orbital shaking at 120 rev. min^-1^ overnight. Cultures were diluted to OD_600_∼0.01 in RPMI 1640 medium and 100 μl aliquots transferred to 96-well microtiter plates (Greiner Bio-One; Stonehouse, United Kingdom) before static incubation for 2 h. Non-adherent cells were removed by three gentle washes with PBS, then 100 μl of fresh RPMI 1640 were added to each well. Plates were incubated at 37°C with orbital shaking at 100 rev. min^-1^ for 24 h. After biofilm formation, biofilms were washed and drugs were added as specified. Cultures were incubated for a further 24 h. The wells were washed three times with PBS and the XTT reaction was performed in YNB by adding XTT to 210 μg.ml^-1^ and menadione to 4.2 μM. After 3 h, 100 μl of the reaction solution was transferred to a new 96-well plate and the absorbance at 490 nm was measured using a BioTek EL800 microplate spectrophotometer.

### Assay of Quinine Uptake

Quinine uptake was assayed by measuring quinine absorbance at 350 nm as described previously (Tindall et al., 2018). Briefly, overnight cultures were diluted to OD_600_∼0.5 in fresh YPD medium and cultured for a further 4 h with shaking. Quinine hydrochloride was added in combination with sodium bicarbonate, hygromycin B or no drug and cells incubated for 20 min at 30°C, 120 rev. min^-1^. Cells were harvested, washed, resuspended in 10% (*w/v*) perchloric acid – 2 M sodium methanesulfonate, and then lysed using acid-washed beads. Supernatant was collected after centrifugation (16,060*g*, 5 min) and *A*_350_ measured with an Ultrospec 2000 UV/visible spectrophotometer (Amersham Pharmacia Biotech; Amersham, United Kingdom).

### Mistranslation Assays

For quantitative determination of mistranslation, *S. cerevisiae* was transformed with a dual luciferase reporter plasmid encoding firefly and *Renilla* luciferases separated by a UAA stop codon (Keeling et al., 2004) and cultured in YNB broth supplemented appropriately for plasmid selection. Cultures were diluted to OD_600_∼0.5 in YPD and 300 μl aliquots transferred to 48-well microtiter plates with compounds added as specified. Plates were incubated at 30°C for 4 h with shaking. Cell extracts were prepared and luciferase activities were measured as described previously (Moreno-Martinez et al., 2015).

## Results

### Discovery of Novel, Synergistic Antifungal Combinations

**FIGURE 1:**
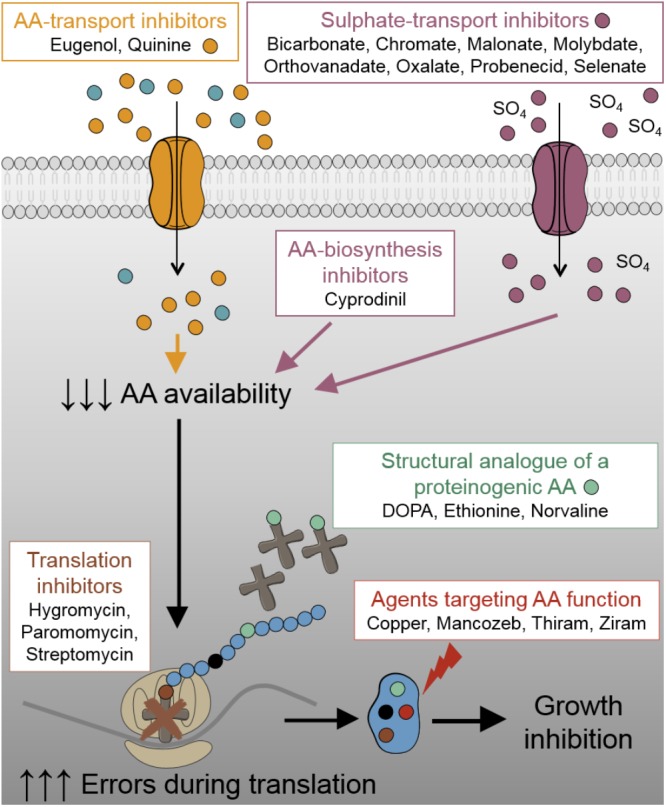
Schematic representation of the different test-agents and their targets in this study. AA, amino acid.

Following recent work suggesting translation fidelity to be a new target for combinatorial treatment of undesirable fungi (Moreno-Martinez et al., 2015), we hypothesized that alternative groups of compounds which compromise the availability of functional amino acids for protein synthesis (**Figure 1**) could offer novel antifungal combinations against this target. As fungal demand for particular amino acids can be satisfied by exogenous supply (e.g., from mammalian serum), which could undermine targeting of amino acid availability, we reasoned that methionine and tryptophan availability could be good candidates in human pathogens; serum levels of these amino acids are low (Tagliamonte et al., 1973; Motil et al., 1994). Furthermore, as cysteine is not an essential amino acid, there is decreased risk that agents targeting cysteine exert toxicity in humans. Therefore, we focused on inhibitors targeting aromatic and sulfur-containing amino acids. Besides sulfate transport inhibitors (chromate, molybdate, orthovanadate, oxalate, malonate, probenecid, bicarbonate, and selenate), which provoke cysteine and methionine starvation (Markovich, 2001; Holland et al., 2010; Moreno-Martinez et al., 2015), we examined amino acid transport inhibitors [quinine and eugenol inhibit aromatic amino acid uptake (Khozoie et al., 2009; Darvishi et al., 2013; Tindall et al., 2018)], amino acid biosynthesis inhibitors [cyprodinil inhibits methionine synthesis (Masner et al., 1994; FRAC, 2018)], non-proteinogenic amino acids [L-DOPA, DL-ethionine, norvaline compete with proteinogenic amino acids (Hartman et al., 2007)], and agents that modify thiol containing amino acids [thiram, ziram, mancozeb (Santos et al., 2009; Dias et al., 2010; Roede and Jones, 2014) and copper; copper additionally targets FeS biosynthesis required for synthesis of certain amino acids (Alhebshi et al., 2012; Vallieres et al., 2017)]. These agents were tested in combination with either: (i) another agent that impairs availability of functional amino acids, or (ii) an aminoglycoside antibiotic. Agents were used at concentrations determined in preliminary experiments to be just subinhibitory (or barely inhibitory) when supplied individually. This design demanded relatively high concentrations of some agents (see section “Discussion”); however, the added volume never exceeded 3% of the final volume (**Table 1**).

**FIGURE 2:**
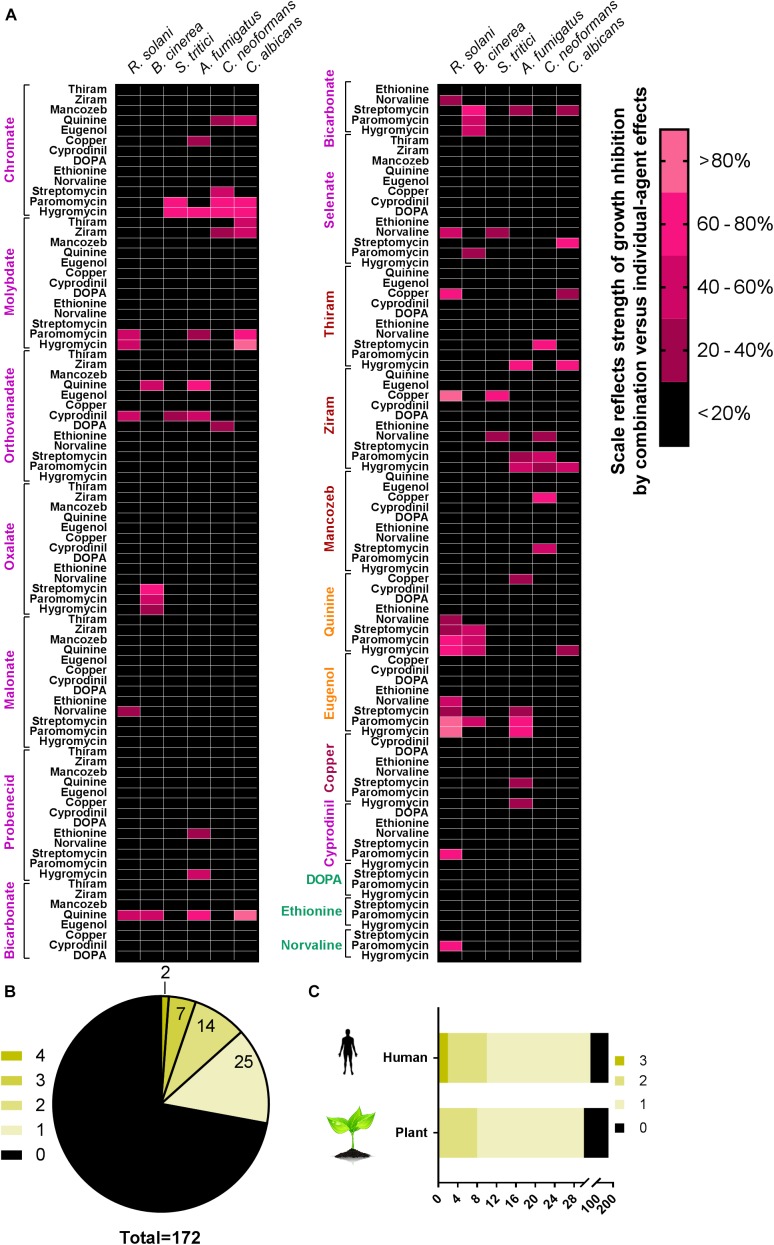
Drug interactions among 172 combinations of agents assayed against six pathogenic fungi. **(A)** For assaying antifungal combinations, fungi were incubated in the presence of just sub-inhibitory or barely inhibitory concentrations of agents supplied individually or in the indicated combinations (the concentrations used are listed and explained in **Table 1**). Growth was measured as described in the section “Materials and Methods.” Combinations showing a >20 percentage-point greater effect of the combination versus the sum of the drugs’ individual % effects were considered screen hits and are represented in pink in the grid (lighter pink = stronger synergistic-type interaction). Compounds are color-coded; each color represents a class described in **Figure 1**. **(B)** Synergistic-like combinations common to different fungi, with the green scale indicating the numbers of the test species affected. **(C)** Synergistic-like combinations common to the different human pathogens or the different plant pathogens.

**FIGURE 3:**
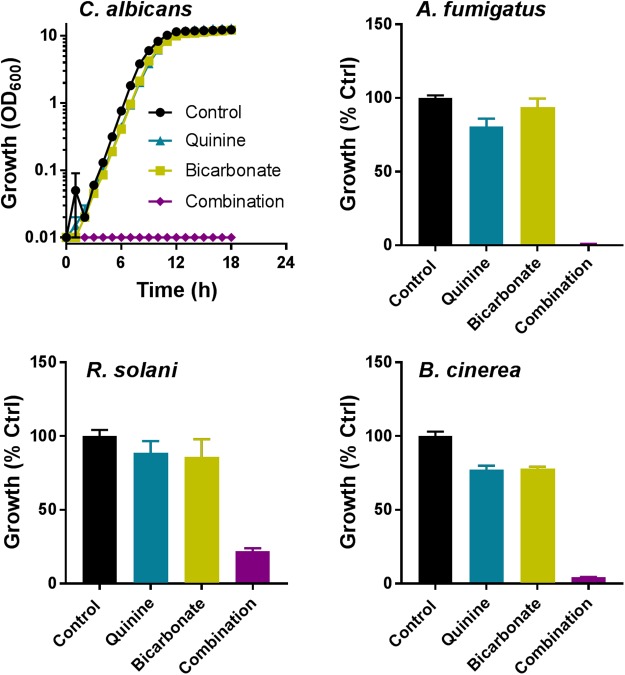
Effect of quinine + bicarbonate on growth of fungal pathogens. Fungi were cultured in the appropriate broth media and OD_600_ determined at intervals. Percentage growth (versus minus drug controls) of the filamentous fungi was determined after 48 h (*A. fumigatus*) or 4–7 days (plant pathogens). Each agent was supplied at its just-subinhibitory concentration for each fungus (**Table 1**). The data are means ± SEM from three independent experiments.

Combination screens were performed against three plant pathogens (ascomycetes *B. cinerea* and *Z. tritici*, and the basidiomycete *R. solani*) and three human pathogens (ascomycetes *C. albicans* and *A. fumigatus*, and the basidiomycete *C. neoformans*). Screen hits were determined as compounds giving a percentage inhibition in combination that was greater by 20% than the sum of inhibitory effects of the compounds when supplied individually, adapted from Foucquier and Guedj (2015). Among the 172 combinations tested, at least one agent from each of the classes targeting different pathways (**Figure 1**) produced synergistic-type inhibition when combined with at least one other agent (**Figure 2A**). Twenty-three of these combinations showed significant inhibition against more than one pathogen (**Figure 2B**). Among these 23, five combinations comprised a sulfate transport inhibitor and an aminoglycoside, with the chromate + hygromycin combination exhibiting synergistic-type inhibition of four of the test fungi. These included *A. fumigatus*, previously shown to resist synergy from chromate with the alternative aminoglycoside paromomycin (Moreno-Martinez et al., 2015), as was confirmed here. Six of the 23 combinations that were effective cross-species involved a tryptophan transport inhibitor (quinine or eugenol) and an aminoglycoside (**Figure 2A**). These combinations did not work against *Z. tritici* and only six of the 172 combinations gave inhibition of this phytopathogen, whereas ≥12 combinations were effective against each of the other tested species. No combination appeared to act synergistically against all three plant pathogens (**Figure 2C**). On the other hand, ziram + hygromycin, like chromate + hygromycin, inhibited all three human pathogens. Generally, combinations involving a dithiocarbamate (ziram or thiram) and an aminoglycoside seemed to target specifically fungal pathogens of humans, and encompassed 3 of the 23 combinations (**Figure 2**). Of those 23, nine did not include an aminoglycoside antibiotic: these were quinine combined with three different sulfate transport inhibitors, orthovanadate + cyprodinil, selenate + norvaline, and a dithiocarbamate with either copper, molybdate, or norvaline (**Figure 2**). The dithiocarbamate + copper combination is used in agriculture. Synergistic-type inhibition by bicarbonate + quinine was observed in four of the six test fungi and so had one of the broadest spectra. This combination conferred very strong growth inhibition, with ∼100% reduction of *C. albicans* growth yield at quinine and bicarbonate concentrations which individually had little effect (**Figure 3**).

Checkerboard analysis was performed against all six pathogens to corroborate synergies for some of the key combinations observed from the screen (**Supplementary Table S2**). Most of the combinations of interest detected from the screen were shown to be synergistic (only 2 exceptions out of 17), with the lowest FIC index values being 0.06 for chromate + hygromycin against *C. albicans* and 0.19 for paromomycin + eugenol (*B. cinerea*) as well as quinine + hygromycin (*R. solani, B. cinerea*). Whereas there were 2 exceptions among hits from the screen, 4 of the checkerboard outcomes indicated synergies that were not detected in the screen, suggesting the screen could be conservative. Three of the outcomes from checkerboards that did not match screen results were for *C. neoformans*, which grew poorly in the static cultures used for the checkerboard assay.

The results revealed a number of combinations that produced synergistic growth inhibition of the fungi. Some of the most effective combinations were tested in the bacterium *P. aeruginosa* and in a human cell line. However, no evidence for synergy was found, suggesting that the synergies may be fungi-specific (**Supplementary Figure S1** and **Supplementary Table S1**). We also tested key combinations against the yeast *S. cerevisiae*, to enable this model’s use for insights to mode of action (below). With the exception of orthovanadate + cyprodinil, the main combinations that produced synergistic-type inhibition against more than two fungal pathogens also did so against *S. cerevisiae*, albeit less markedly in the case of ziram + hygromycin (**Supplementary Figure S2**). Regarding combinations of an aromatic amino acid transport inhibitor with an aminoglycoside, the data suggested a stronger synergistic effect of quinine + hygromycin than eugenol + paromomycin on *S. cerevisiae*.

### Synergies Involving Quinoline-Containing Drugs Have Fungicidal and Anti-biofilm Actions

**FIGURE 4:**
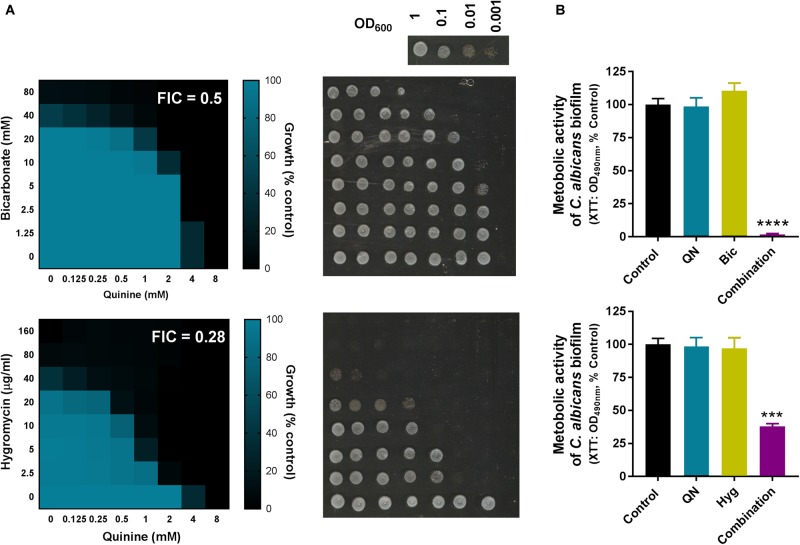
Synergistic quinine combinations are fungicidal and biofilm active. **(A)** Checkerboard assays according to EUCAST procedure, performed in YPD broth with *S. cerevisiae* BY4743 at the indicated concentrations of quinine and bicarbonate or hygromycin. The growth values are percentages of growth (OD_600_) with the agents relative to the minus-drug control. FIC, fractional inhibitory concentrations calculated from the data (Hsieh et al., 1993). After 24 h growth with the specified checkerboard drug concentrations, cell suspensions from the checkerboard assays were spotted onto YPD agar without drugs and incubated at 30°C for 24 h before plates were imaged to assess colony forming ability. The inset at top right shows control growth from cell suspensions prepared at different dilutions; the OD_600_∼0.1 spot corresponds to growth at the starting-inoculum density used for the checkerboards (i.e., the growth anticipated if agents were fungi static rather than fungi cidal). **(B)** Mature *C. albicans* biofilms were exposed to 2 mM quinine (QN) and/or 40 mM bicarbonate (Bic) or 7.5 μg/ml hygromycin (Hyg) for 24 h. Metabolic activity of biofilms was assessed using XTT. ^∗∗∗∗^*p* < 0.0001 and ^∗∗∗^*p* < 0.001 according to multiple comparisons (Tukey’s test) by two way ANOVA. The values are means ± SEM from three independent experiments.

Several of the most effective combinations in the above screens involved quinine, with quinine + hygromycin and quinine + bicarbonate appearing especially effective cross-species (**Figure 2A**). The antimalarial quinine is a food and drug approved agent, like hygromycin and bicarbonate, so these novel combinations provided a good focus for further investigation. Resistance erodes the efficacy of current antifungal drugs, but resistance is typically less common against fungicidal than fungistatic drugs (Anderson, 2005). To determine whether the combinations with quinine were fungicidal or fungistatic, we set up checkerboard growth assays with the drugs followed by transfer to drug-free control medium to assess retention of colony forming ability, using the yeast model. The data revealed that both of the quinine combinations, with either bicarbonate or hygromycin, were fungicidal: cells exhibiting growth inhibition during incubation in the presence of compounds were unable to form colonies when recovered to the control medium (**Figure 4A**).

*Candida albicans* was among the pathogens synergistically inhibited by the quinine + bicarbonate/hygromycin combinations. This organism’s ability to form drug-resistant biofilms has been associated with high mortality rates in infected, immunocompromised patients (Silva et al., 2017; Van Dijck et al., 2018). We tested the effect of both of the new combinations against fungal metabolic activity within biofilms. In both cases, biofilm metabolic activity was decreased by >60% with the combinations, at doses of each agent which had no effect individually (**Figure 4B**). Therefore, the synergy was effective against *C. albicans* within biofilms.

**FIGURE 5:**
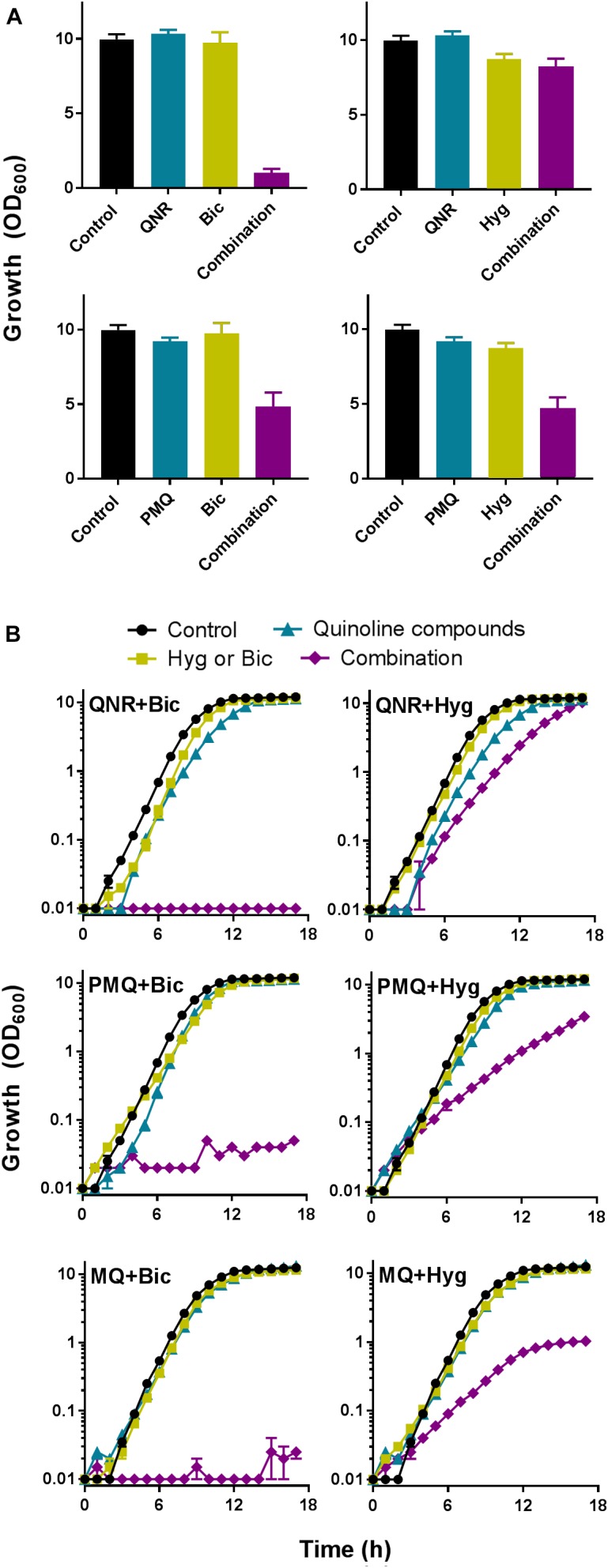
Bicarbonate and hygromycin exhibit synergistic interactions with several quinoline antimalarial drugs. **(A)** *B. cinerea* was cultured in PDB supplemented with each indicated agent supplied at a concentration that was just-subinhibitory or barely inhibitory (**Table 1**). OD_600_ was measured after 5 days. QNR, quinacrine; PMQ, primaquine; Bic, bicarbonate; Hyg, hygromycin (*B. cinerea* was not tested with mefloquine, MQ). **(B)** *C. albicans* was cultured in YPD with indicated agents at the relevant concentrations listed in **Table 1**. The values are means ± SEM from three replicate experiments.

**FIGURE 6:**
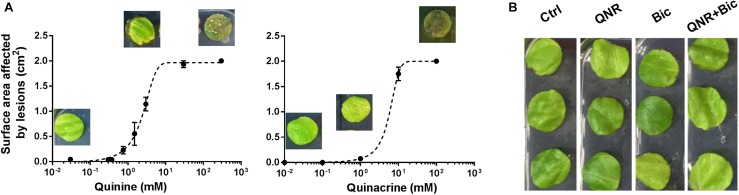
Quinine and quinacrine show little toxicity *in planta*. Agents were sprayed onto lettuce leaves which were examined after 3 days for lesions arising from quinine or quinacrine individually **(A)**, and quinacrine in combination with bicarbonate **(B)**. The images in panel **(A)** correspond to the relevant, indicated drug concentrations. Total surface area of the leaf disks was 2 cm^2^. The values are means ± SEM from eight leaves. Ctrl, control; QNR, 100 μM quinacrine; Bic, 20 mM bicarbonate.

Quinine is one of several quinoline-derived antimalarial drugs. We hypothesized that antifungal synergy with bicarbonate or hygromycin may not be restricted to quinine, so tested different examples of these agents for synergistic interactions against representative plant and human pathogens (*B. cinerea* and *C. albicans*). In both organisms, quinacrine gave strong synergistic-type inhibition in combination with bicarbonate, but not hygromycin. Primaquine and mefloquine exhibited synergy with both compounds, particularly bicarbonate (**Figure 5**). Quinoline-derived compounds are used in medicine, but not yet in agriculture and plant toxicity might be an issue. Therefore, we tested for effects of the agents on leaf material, from lettuce. The compounds were sprayed at different concentrations onto leaves and the average surface area of any arising lesions determined. Treating the leaves with quinine or quinacrine, at the concentrations used above against the plant pathogen (i.e., 1 mM quinine and 100 μM quinacrine), produced negligible damage (lesions) (**Figure 6A**). The subtoxicity concentrations *in planta* of bicarbonate and hygromycin were also determined (not shown), according to doses that produced <10% surface-area lesions; these concentrations were respectively, 5 and 50 times higher than the subinhibitory concentrations used against *B. cinerea*. We tested whether quinacrine + bicarbonate (the most effective combination against *B. cinerea*) had a synergistic effect on plant material, at the same concentrations used against *B. cinerea*. No leaf lesions were observed (**Figure 6B**). In conjunction with the data for bacterial and human cells (**Supplementary Figure S1** and **Supplementary Table S1**), the data indicated that synergistic inhibition by the combinations involving quinoline-derived compounds was selective for fungi.

### Mechanisms of Synergistic Action

**FIGURE 7:**
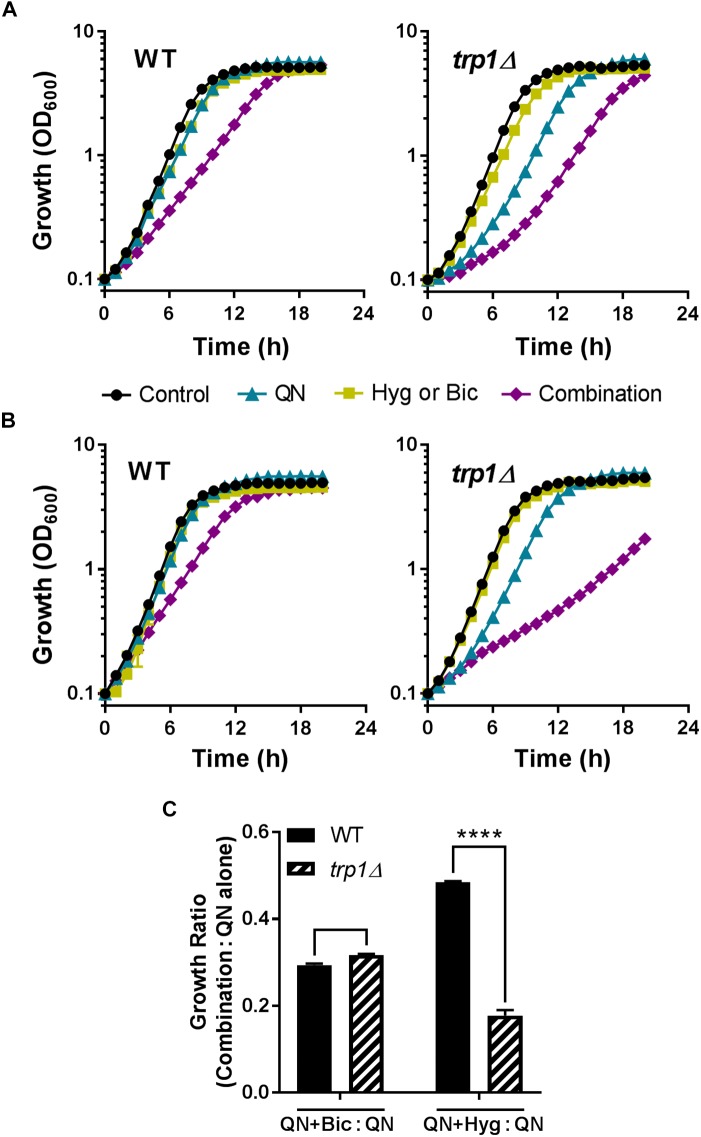
Tryptophan depletion can exacerbate quinine + hygromycin synergy. *S. cerevisiae* BY4743 and *trp1*Δ** strains were cultured in either the absence or presence of 2 mM quinine hydrochloride (QN) and either sodium 6 mM bicarbonate (Bic) **(A)** or 3 μg/ml hygromycin B (Hyg) **(B)**. **(C)** Growth ratios (combination:QN alone) were calculated from OD_600_ values at 10 h. ^∗∗∗∗^*p* < 0.0001 according to multiple comparisons (Sidak’s test) by two way ANOVA. The values are means ± SEM from three replicate experiments.

The hypothesized mode of action of the combinations was perturbation of mRNA translation fidelity, via amino acid depletion. The principal mode(s) of quinine drug action is unresolved, but one effect detected first in the yeast model is that quinine competes with tryptophan to cause tryptophan starvation (Khozoie et al., 2009; Tindall et al., 2018). To test a role for tryptophan here, we used a *S. cerevisiae trp1*Δ** mutant defective for tryptophan biosynthesis, so reliant on uptake of the amino acid from the growth medium. We reasoned that this strain should be hyper-sensitive to a synergistic action which involves tryptophan starvation. As observed previously (Khozoie et al., 2009; Tindall et al., 2018), the auxotrophic *trp1*Δ** mutant was sensitive to quinine alone (only a small sensitization was observable at the quinine concentration used here) (**Figures 7A,B**). However, *trp1*Δ** cells were no more sensitive to the quinine + bicarbonate combination, relative to quinine alone, than the wild type (**Figures 7A,C**). In contrast, the *trp1*Δ** mutant was hyper-sensitive to the quinine + hygromycin combination (**Figures 7B,C**). This suggested that tryptophan depletion in conjunction with quinine exposure exacerbates the action of hygromycin specifically.

**FIGURE 8:**
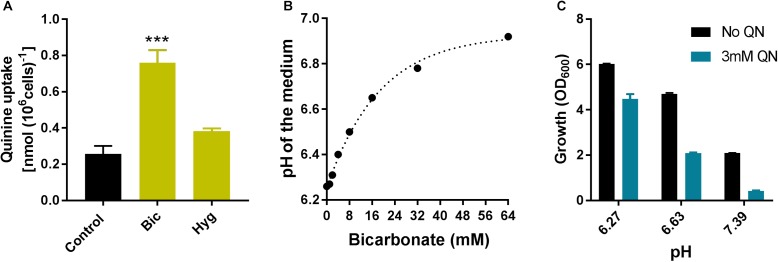
Bicarbonate increases quinine uptake. **(A)** Wild type yeast cells were incubated for 20 min with 4 mM quinine in combination or not with 7.5 mM bicarbonate (Bic) or 10 μg/ml hygromycin (Hyg) [20 min is the approximate mid-point of quinine accumulation by yeast; (Tindall et al., 2018)]. Quinine determinations in cell lysates according to *A*_350_ were normalized for cell concentration measured just before lysis, with subtraction of background determinations for cells incubated without quinine. ^∗∗∗^*p* < 0.001 according to multiple comparisons (Tukey’s test) by two way ANOVA. The values are means ± SEM from three independent experiments. **(B)** The pH of YPD medium was determined following additions of different amounts of sodium bicarbonate to give the indicated final concentrations. **(C)** Wild type yeast was incubated in the presence or absence of 3 mM quinine in YPD medium adjusted to different pH values by NaOH addition. Growth (OD_600_) was measured after 9 h. The values are means ± SEM from three independent experiments.

Cellular quinine levels were assayed to determine whether bicarbonate or hygromycin may alter drug uptake, an alternative potential explanation for the synergies. Hygromycin did not have a significant effect on quinine uptake. In contrast, cells incubated with bicarbonate showed ∼3-fold greater quinine uptake (**Figure 8A**). Bicarbonate additions can make media more alkaline (the media used here were unbuffered) and we observed in a previous study that NaOH had a stimulatory effect on quinine uptake (Tindall et al., 2018). The bicarbonate concentration used in the combination treatments (7.5 mM) increases the pH of YPD medium from 6.25 to ∼6.5 (**Figure 8B**), which is sufficient to explain some exacerbation of growth inhibition by quinine: an increase of ∼0.36 pH units increased quinine-dependent growth inhibition from ∼25% to >55% relative to control growth at the same pH values (**Figure 8C**). The results show that stimulation of growth-inhibitory quinine action by bicarbonate is correlated with increased quinine uptake, and may be related to medium alkalinization by bicarbonate.

**FIGURE 9:**
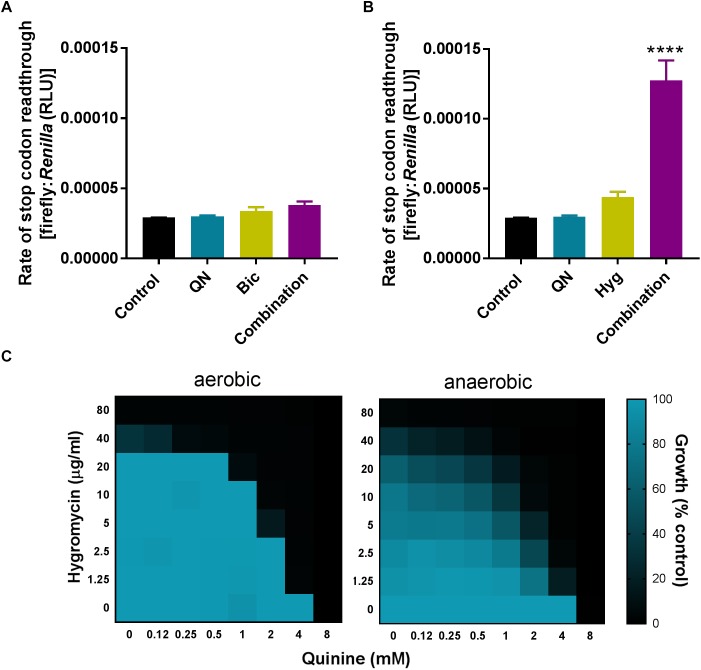
Quinine synergistically increases mistranslation rate when combined with hygromycin but not bicarbonate. **(A,B)** *S. cerevisiae* transformed with a dual-luciferase plasmid encoding the *Renilla* and firefly luciferases separated by a UAA stop codon was exposed to 3 mM quinine (QN), 7.5 mM bicarbonate (Bic), or 2.5 μg/ml hygromycin (Hyg) alone or in combination for 4 h, before determination of the two luciferase activities. ^∗∗∗∗^*p* < 0.0001 according to multiple comparisons (Tukey’s test) by two way ANOVA. The values are means ± SEM from three independent experiments. **(C)** Checkerboard assays according to EUCAST procedure, performed in YPD broth with *S. cerevisiae* BY4743 at the indicated concentrations of quinine and hygromycin in aerobic and anaerobic conditions. The growth values are percentages of growth (OD_600_) with the agents relative to the minus-drug control.

To test whether synergistic growth inhibition was reflected by synergistic mistranslation, we used a quantitative mistranslation assay. The assay tests for inappropriate readthrough of a UAA stop codon, positioned between ORFs encoding *Renilla* and firefly luciferases. The firefly luciferase should only be expressed following UAA readthrough, so the ratio of the luciferase activities reflects the rate of mistranslation. The quinine + bicarbonate combination did not increase the rate of stop codon readthrough (**Figure 9A**). However, readthrough was increased ∼4-fold by a combination of quinine + hygromycin, where neither agent alone had a strong effect at the doses used (**Figure 9B**).

Quinoline-containing compounds are known to provoke reactive oxygen species (ROS) production (Radfar et al., 2008; Tafazoli and O’Brien, 2009; Laleve et al., 2016). As ROS may in turn promote oxidation of RNA or of proteins involved in translation (Ling and Soll, 2010; Vallieres and Avery, 2017b), alternative potential causes of mistranslation, we tested whether the synergy between quinine + hygromycin (associated with elevated mistranslation) could be suppressed by the absence of oxygen. However, synergy was slightly enhanced rather than rescued under anaerobic growth conditions (**Figure 9C**). The absence of rescue by anaerobicity leaves open the starting hypothesis: that amino acid limitation by quinine (rather than ROS generation) could be the relevant mechanism exacerbating mistranslation caused by hygromycin. In contrast, we showed that bicarbonate enhanced quinine uptake (and resultant growth inhibition), without stimulating stop codon readthrough. The data indicate that the different combinations involving quinine could exert different modes of synergistic antifungal action.

## Discussion

Previously, it was reported that combination of a sulfate transport inhibitor with an aminoglycoside synergistically inhibits growth of several fungi (Moreno-Martinez et al., 2015). The present results establish that diverse compounds which can alter amino acid availability in different ways, exert synergistic antifungal inhibition when combined with other agents impairing amino acid availability or with aminoglycosides. Despite these common features of the agents tested, the mechanism of synergistic action is not the same for every combination. Moreover, the dataset greatly expands this repertoire of synergistic interactions affecting fungal pathogens of both plants and humans.

Amino acid biosynthesis has been described as an important target of antifungal agents (Jastrzebowska and Gabriel, 2015). Numerous studies have shown that auxotrophic mutants of fungi pathogenic to humans, impaired in biosynthesis of particular amino acids, exhibit growth defects or at least reduced virulence *in vivo*. Furthermore, chemicals targeting amino acid biosynthetic enzymes can exert good antifungal activity *in vitro* (Jastrzebowska and Gabriel, 2015). Inhibitors of such fungal enzymes had little if any toxicity in mammalian systems and were not subject to fungal multidrug resistance. Depletion of particular amino acids within cells can alter the competition between cognate and non-cognate aminoacyl-tRNAs, leading to mRNA mistranslation (Gallant and Lindsley, 1998; Farabaugh and Bjork, 1999; Sorensen, 2001). Perturbation of translation fidelity may be a particularly attractive antifungal action as it not only causes loss of (essential) protein functions, but also gain of toxic function through formation of inhibitory protein aggregates (Holland et al., 2007). Aminoglycosides are thought to promote translation errors by causing a structural alteration in the small ribosomal subunit decoding center, allowing entry of near-cognate tRNAs (Carter et al., 2000; Fan-Minogue and Bedwell, 2008). In the case of combinations with aminoglycosides [e.g., aminoglycoside combined with a sulfate- (Moreno-Martinez et al., 2015) or tryptophan- (this study) transport inhibitor], synergistic growth effects were accompanied by synergistic increases in translation error-rate. This could be explained by the dual targeting of translation fidelity, through different mechanisms, by each agent in the combinations. The absence of synergy against bacteria, a human cell line or plant leaves, reported here, could arise if the effect of at least one agent in a combination is fungus specific. For instance, methionine or cysteine supply in human cells is from the diet and not biosynthesis requiring sulfate uptake. Therefore, sulfate-mimetics (or indeed many other inhibitors of amino acid biosynthesis) should not provoke mistranslation as in fungi (Holland et al., 2010).

A decreased risk of resistance evolution against synergistic combinations of agents should be especially marked when agents have fungicidal rather than fungistatic effects (Anderson, 2005; Brent and Hollomon, 2007; Hill et al., 2015). Fungi treated with fungistatic drugs like azoles, used widely in medicine and agriculture, are in constant contact with the agents while the organism remains viable, increasing the opportunity for resistance emergence. In contrast, the combinations involving quinine reported here had fungicidal activity. Additional features of quinine that are of interest here were its antifungal synergy in combination with either bicarbonate or hygromycin, the efficacy of these synergies against *C. albicans* biofilms, and that all three compounds are food and drug approved, meaning they could be re-purposed more readily than novel agents due to the low development risk. One limitation could relate to the apparent drug concentrations needed to control fungal growth, as addressed in the next paragraph. One pertinent point concerning quinine is that common quinine analogs, albeit typically more expensive than quinine, can have much lower effective MICs against fungi (**Table 1**; Vallieres and Avery, 2017b).

Our experimental design, to supply drugs at concentrations which individually were just subinhibitory, demanded concentrations of some agents which were higher than could be realistic for therapeutic use in humans or for large scale crop treatments. However, it should be borne in mind that the design was intended to give the most sensitive detection for discovering synergistic interactions, rather than replicate *in vivo* systems, and that synergies persist at considerably lower concentrations than we selected. Furthermore, rich fungal media can dampen activities of different inhibitors compared with human serum or fungicide formulations, e.g., by complexation of agents with medium components (Hughes and Poole, 1991) or, particularly pertinent to this study, high availability of amino acids in media, e.g., cysteine and tryptophan (Khozoie et al., 2009; Holland et al., 2010; Tindall et al., 2018). We have also shown that cysteine or tryptophan limitation can mimic the effects of synergistic drug combinations with aminoglycosides, for example (**Supplementary Figure S3**). Furthermore, we showed that medium pH can markedly influence quinine toxicity. The high-bicarbonate, high-pH nature of DMEM used for human cell culture could explain the relative quinine-susceptibility apparent in TE671 cells, for example (**Table 1**). Finally, as discussed further below, the present data provide proof of principle for novel synergistic interactions which provide a basis for rational formulation of more effective combinations, if needed, centered around similar chemistries.

We had hypothesized that tryptophan starvation arising from competition between quinine and tryptophan for uptake (Khozoie et al., 2009; Tindall et al., 2018) would stimulate mistranslation in combinations of quinine with bicarbonate (sulfate mimetic) or hygromycin (aminoglycoside). The hyper-sensitivity to synergistic inhibition by quinine + hygromycin that we observed in a *trp1*Δ** yeast mutant supported this, as did synergistic stimulation of mistranslation by this drug combination. In contrast, our observation that bicarbonate increased quinine uptake could alone account for that synergy as increased intracellular quinine should increase quinine toxicity. The alkalinizing effect of bicarbonate could impact quinine uptake though altered membrane energetics, for example. Moreover, collectively the data indicate that some but not all of the synergies observed here may be underpinned by translation-error, the starting hypothesis which originally informed our choice of agent-classes to test. Certainly several of the test agents will, like bicarbonate, have effects on cells additional to perturbation of translation fidelity. Given that, it will be interesting to resolve mechanism for a broader range of the new synergistic combinations discovered here.

The novel combinations of this study could open opportunities for control of fungal pathogens. The list of agents screened within each class was not exhaustive, so the most promising combinations (e.g., quinine + bicarbonate and quinine + hygromycin) could be used as a basis for computational chemistry and other rational approaches to further optimize properties and synergies. In addition, while we focused primarily on agents targeting sulfur-containing amino acids and aromatic amino acids, it is likely that agents affecting availability of other amino acids may also produce synergies. For example, inhibitors of the NADP- and NAD-glutamate dehydrogenases are known to be active against fungi such as *Aspergillus niger* and *C. albicans* (Choudhury et al., 2008; Joshi et al., 2013) and could provide one group of promising candidates for additional synergistic effects.

None of the combinations were synergistic against all six of the fungal pathogens tested. The overall pattern of susceptibility to the different combinations did not map clearly to phylogenetic relatedness of the fungi. For example, whereas 23 combinations strongly inhibited more than one fungus, none were effective against both of the basidiomycetes (*R. solani* and *C. neoformans*). Also, with certain exceptions, there was no consistent relationship within the human or plant pathogens. Drug resistance phenotypes can arise from a resistant (or absent) molecular target, reduced drug uptake, increased efflux, or drug inactivation, modification or sequestration. In addition, resistance of certain fungi to certain drugs was so high (e.g., *C. albicans* with streptomycin) that just-subinhibitory concentrations were not attained for some synergy assays. It is possible that synergy may become detectable at higher concentrations in such cases. The different test-fungi are cultivated on different media, where contents of amino acids, sulfate and other relevant substrates differ, potentially impacting the phenotypes reported here. Previously, it was reported that *B. cinerea* was not susceptible to synergistic inhibition by chromate + paromomycin (Moreno-Martinez et al., 2015). However, in the present study, several alternative sulfate mimetics (oxalate, bicarbonate, and selenate) did synergistically inhibit *B. cinerea* when combined with aminoglycosides. This emphasizes the need, where possible, to assess a range of agents associated with a similar mode of action.

## Conclusion

In conclusion this work unveils a panel of novel chemical combinations, of agents affecting amino acid availability or translation, which synergistically inhibit fungal pathogens. Besides the therapeutic potential for exploitation of these new insights, some of these activities could provide valuable tools for understanding the control of translation fidelity in fungi, and how it may unravel.

## Data Availability

Most of the relevant data are included within the manuscript and **Supplementary Files**. Any additional raw data supporting the conclusions will be made available by the authors on request, without undue reservation, to any qualified researcher.

## Author Contributions

SA and CV devised the study and drafted the manuscript. CV and RR performed the experiments. SA and MD provided resources and intellectual input which supported the study.

## Conflict of Interest Statement

The authors declare that the research was conducted in the absence of any commercial or financial relationships that could be construed as a potential conflict of interest.
