# Supplementary material for: Novel Combinations of Agents Targeting Translation That Synergistically Inhibit Fungal Pathogens
Source: Front Microbiol. 2018 Oct 4;9:2355. doi: 10.3389/fmicb.2018.02355 (PMC6186996; doi:10.3389/fmicb.2018.02355)
Supplement: Supplementary file 1 [file Data_Sheet_1.PDF]

SUPPLEMENTAL MATERIAL
Vallières et al.

Table S1 Weak effects of combinations against human TE671 cells. Human TE671 cells were cultured in DMEM supplemented with just-subinhibitory concentrations of agents (Table 1). Cytotoxicity effects of the combinations were determined using CCK-8 reagent as described in the methods. The values show relative metabolic activity (formazan production as a percentage of that in the minus drug control) and are means ± SEM from three replicate determinations.

Agents (A + B)	% Metabolic activity		
	Agent A	Agent B	Agent A + B
Quinine + Bicarbonate	84.90 ±0.04	108.47 ±0.01	71.39 ±0.02
Quinine + Hygromycin	84.00 ±0.00	91.01 ±0.00	68.87 ±0.01
Eugenol + Paromomycin	98.51 ±0.02	90.42 ±0.01	96.59 ±0.01
Orthovanadate + Cyprodinil	83.25 ±0.06	90.82 ±0.02	71.45 ±0.03
Selenate + Norvaline	93.32 ±0.00	95.34 ±0.03	88.82 ±0.01
Ziram + Copper	78.67 ±0.08	74.31 ±0.01	77.76 ±0.02
Ziram + Hygromycin	101.61 ±0.01	89.71 ±0.01	84.03 ±0.01

Table S2 FIC indices for main combinations of interest. Checkerboard assays were performed as described in the Materials and Methods section. Synergy was defined by FIC index ≤ 0.5. FIC indices between 0.5 and 1 were interpreted as additive, and between 1 and 4 as indifference. Pink shading indicates combinations that were deemed hits for the relevant fungi from the initial screening (Figure 2).

	R. solani	***B. cinerea***	***S. tritici***	***A. fumigatus***	***C. neoformans***	***C. albicans***
Chromate + Hygromycin	0.63	0.51	0.25	0.5	0.19	0.06
Quinine + Bicarbonate*	0.38	0.5	0.19	0.25*	0.5	0.53*
Paromomycin + Eugenol	0.38	0.19	0.38	0.26	0.38	0.51
Ziram + Hygromycin	>1	1	>1	0.31	1	0.37
Quinine + Hygromycin	0.19	0.19	>1	>1	0.51	0.5

*the bicarbonate MIC could not reliably be reached for *A. fumigatus* and *C. albicans*, meaning that these FICI values may be overestimates.

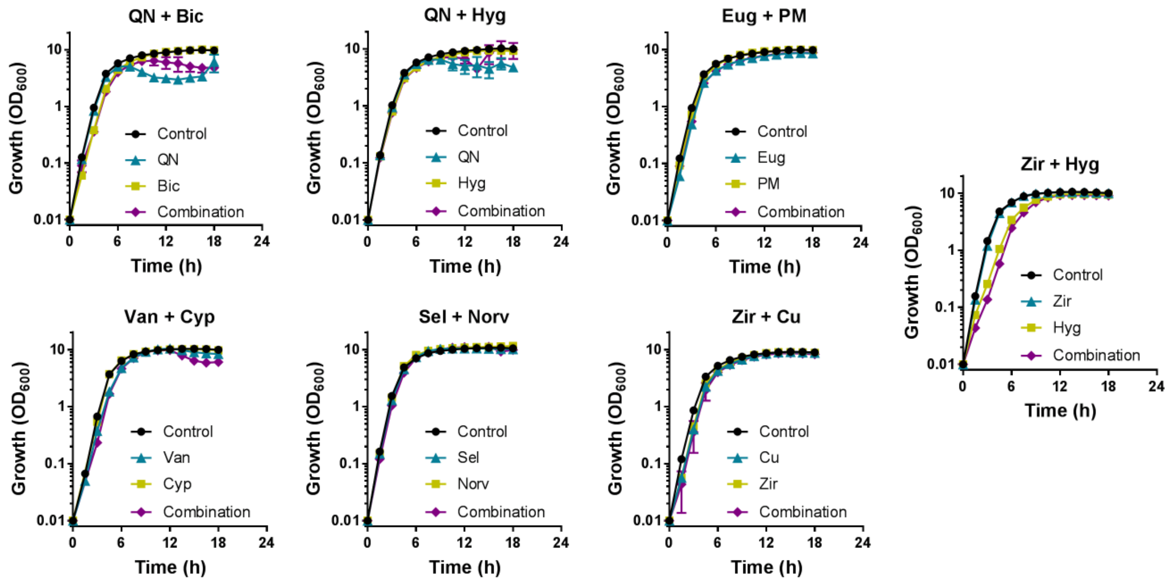

FIGURE S1 Absence of synergy against *Pseudomonas aeruginosa*. Bacteria were cultured in LB medium supplemented with agents alone (supplied at just-subinhibitory or barely inhibitory concentrations, as listed in Table 1) or in combination. The values are means ± SEM from three independent experiments. QN, quinine; Bic, bicarbonate; Hyg, hygromycin, Eug, eugenol; PM, paromomycin; Van, Orthovanadate; Cyp, cyprodinil; Sel, selenate; Norv, norvaline; Zir, ziram; Cu, copper.

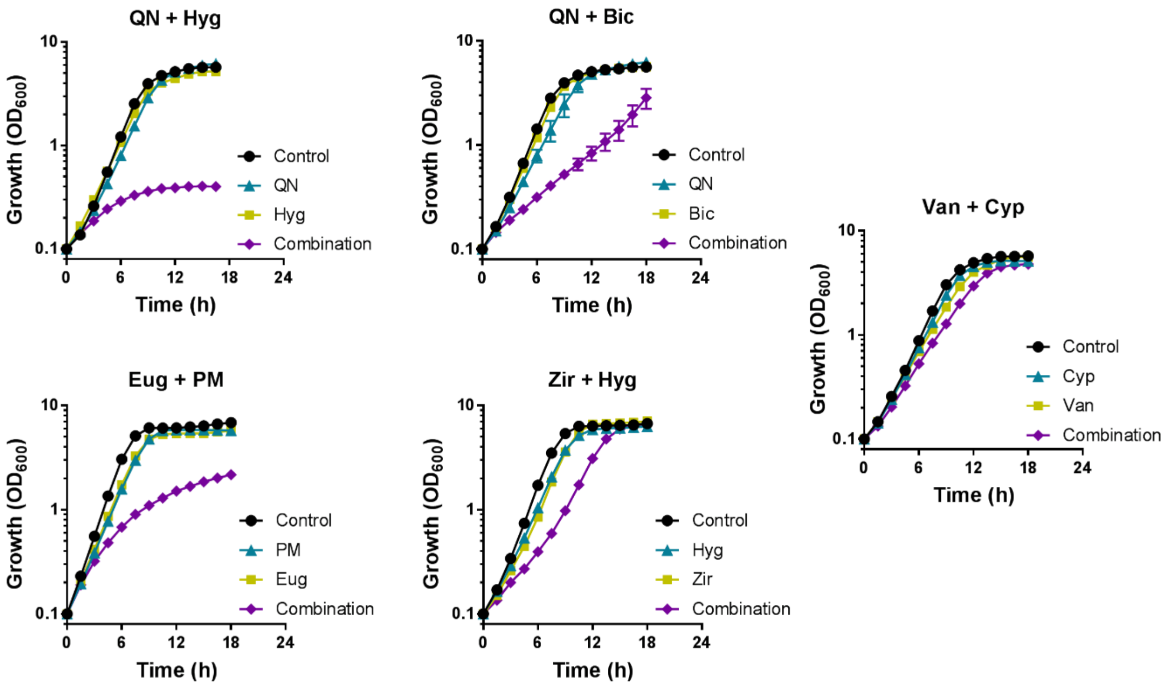

FIGURE S2 Effect of drug combinations of interest against *Saccharomyces cerevisiae*. Yeast were cultured in YPD supplemented with agents alone (supplied at just-subinhibitory or barely inhibitory concentrations) or in combination. The values are means ± SEM from three independent experiments. QN, 3 mM quinine; Bic, 7.5 mM bicarbonate; Hyg, 10 µg/ml hygromycin; Eug, 600 µM eugenol; PM, 200 µg/ml paromomycin; Van, 1.5 mM orthovanadate; Cyp, 50 µM cyprodinil; Zir, 750 ng/ml ziram.

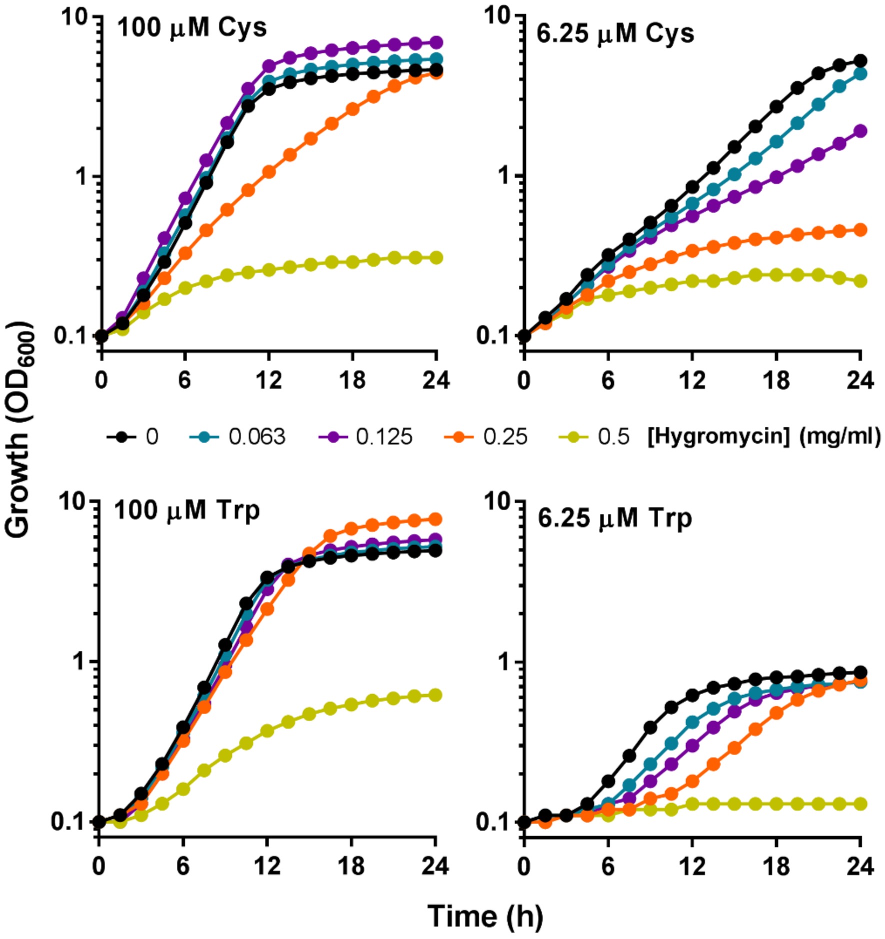

FIGURE S3 Growth inhibition by hygromycin is accentuated in cysteine- or tryptophan-limited cultures. *S. cerevisiae* deletion-strains *cys3Δ* (top row) or *trp1Δ* (bottom row), auxotrophs for cysteine or tryptophan respectively, were cultured in YNB amended to contain limiting (6.25 μM) or non-limiting (100 μM) concentrations of cysteine or tryptophan, and supplemented with the indicated concentrations of hygromycin. Growth inhibition by hygromycin relative to the corresponding minus-drug controls was accentuated in the amino acid limited cultures.
